# Review of methods and antimicrobial agents for susceptibility testing against *Pythium insidiosum*

**DOI:** 10.1016/j.heliyon.2020.e03737

**Published:** 2020-04-12

**Authors:** Hanna Yolanda, Theerapong Krajaejun

**Affiliations:** a Section for Translational Medicine, Faculty of Medicine, Ramathibodi Hospital, Mahidol University, Bangkok, Thailand; b Department of Parasitology, School of Medicine and Health Sciences, Atma Jaya Catholic University of Indonesia, Jakarta, Indonesia; c Department of Pathology, Faculty of Medicine, Ramathibodi Hospital, Mahidol University, Bangkok, Thailand

**Keywords:** Microbiology, Pythiosis, *Pythium insidiosum*, Susceptibility, Antimicrobial agent

## Abstract

Pythiosis is a life-threatening infectious disease of humans and animals caused by the oomycete microorganism *Pythium insidiosum*. The disease has been increasingly diagnosed worldwide. *P. insidiosum* inhabits freshwater and presents in two forms: mycelium and zoospore. Clinical manifestations of pythiosis include an infection of the artery, eye, skin, or gastrointestinal tract. The management of pythiosis is problematic due to the lack of effective treatment. Many patients die from an uncontrolled infection. The drug susceptibility testing provides clinically-useful information that could lead to proper drug selection against *P. insidiosum*. Currently, no standard CLSI protocol for the drug susceptibility of *P. insidiosum* is available. This review aims at describing methods and antimicrobial agents for susceptibility testing against *P. insidiosum*. Several in-house *in vitro* susceptibility methods (i.e., broth microdilution method, radial growth method, and agar diffusion method) have been established for *P. insidiosum*. Either mycelium or zoospore can be an inoculum. Rabbit is the commonly-used model of pythiosis for *in vivo* drug susceptibility testing. Based on the susceptibility results (i.e., minimal inhibitory concentration and inhibition zone), several antibacterial and antifungal drugs, alone or combination, exhibited an *in vitro* or *in vivo* effect against *P. insidiosum*. Some distinct compounds, antiseptic agents, essential oils, and plant extracts, also show anti-*P. insidiosum* activities. Successfully medical treatment, guided by the drug susceptibility data, has been reported in some pythiosis patients. Future studies should emphasize finding a novel and effective anti-*P. insidiosum* drug, standardizing *in vitro* susceptibility method and correlating drug susceptibility data and clinical outcome of pythiosis patients for a better interpretation of the susceptibility results.

## Teaser

Immunology; Microbiology; Pythiosis; *Pythium insidiosum*; Susceptibility; Antimicrobial agent

## 1 Introduction

Pythiosis is a life-threatening infectious disease of humans and animals caused by the oomycete microorganism *Pythium insidiosum* [1, 2, 3]. Pythiosis has been increasingly diagnosed worldwide [4, 5, 6, 7, 8, 9, 10, 11, 12, 13, 14, 15, 16, 17, 18, 19, 20, 21, 22, 23, 24, 25]. The disease affects various mammals, predominantly humans [6, 7, 8, 9, 10, 19, 20], horses [14, 18, 26, 27], and dogs [21, 28, 29]. *P. insidiosum* inhabits freshwater and presents in two forms: mycelium and zoospore [30, 31, 32]. Clinical manifestations of pythiosis include an infection of artery, eye, skin, or gastrointestinal tract [33, 34, 35, 36, 37, 38, 39, 40, 41, 42, 43, 44, 45, 46, 47, 48, 49, 50]. Pythiosis exhibits high morbidity and mortality rates [7, 8, 28, 49, 51]. Early diagnosis and prompt treatment are critical factors to determine the favorable outcome of an affected individual. The diagnosis of pythiosis relies on clinical presentation and laboratory investigations [10, 52, 53, 54, 55, 56, 57, 58, 59, 60, 61, 62, 63, 64, 65, 66, 67, 68]. The use of conventional antifungal drugs is usually ineffective against *P. insidiosum* due to the lack of a drug-target ergosterol biosynthesis pathway [31, 69]. The main treatment of pythiosis in humans and animals (including equine, the most affected species) relies on extensive surgical intervention [14, 26, 70, 71, 72, 73, 74, 75]. Such treatment is expensive and could lead to postsurgical complications and life-long disability. Many patients die from an uncontrolled infection [76, 77, 78]. A more effective treatment is urgently needed for pythiosis.

Many investigators have searched for a chemical that is capable of inhibiting *P. insidiosum* [79, 80, 81, 82, 83, 84, 85]. Although a standardized susceptibility method for *P. insidiosum* is not available, several *in vitro* and *in vivo* assays have been proposed to evaluate drugs against the pathogen [86, 87, 88, 89]. Here, we summarized recent advances in anti-*P. insidiosum* agents and the in-house susceptibility methods for testing them. Such information could facilitate the selection of the most suitable and effective drug for the treatment of pythiosis. This work was approved by the Committee for Research, Faculty of Medicine Ramathibodi Hospital, Mahidol University (Approval numbers: MURA2019/713, MURA2019/1227, and MURA2020/291).

## 2 Drug susceptibility testing against *P. insidiosum*

### 2.1 Inoculum preparation

#### 2.1.1 Zoospores

Zoospore is an infective stage of *P. insidiosum* and colonizes on a water plant. Upon exposure to a human or animal host, the zoospore attaches and germinates as hyphae into affected tissue [31]. Zoospores can be prepared in a laboratory and used as an inoculum for *in vitro* or *in vivo* susceptibility testing against *P. insidiosum* [79, 86, 90, 91]. The method for the production of zoospores is described in detail elsewhere [91, 92, 93]. Briefly, *P. insidiosum* is induced to produce zoospores by co-incubation with sterile grass leaves (i.e., *Paspalum notatum*) on 2% water agar (pH 6.9) at 37 °C for 24 h. *P. insidiosum*-colonizing grass leaves are immersed in the induction medium. After incubation at 37 °C for a few hours, a zoosporangium, containing up to 40 mobile biflagellate zoospores, can be observed under a microscope [92]. Released zoospores can swim ~25 min before encystment [92]. The zoospores are collected, counted by a Neubauer chamber [91, 93], and adjusted to 2–3 x 10^3^ cells/ml in RPMI for a drug susceptibility assay [86, 90].

#### 2.1.2 Mycelia

Two types of mycelial inoculum can be prepared: hyphal suspension and agar plugs. For the hyphal suspension, *P. insidiosum* is subcultured on 0.1% yeast extract agar and incubated at 37 °C for four days [94]. The colony is then scraped using a sterile scalpel blade in the presence of 10 ml sterile distilled water [94, 95]. The obtained hyphal suspension is adjusted to 80–85% transmittance at the 530-nm wavelength. The hyphal suspension is diluted (1:10) in the Roswell Park Memorial Institute (RPMI) 1640 broth before using it as inoculum in the broth microdilution method [94, 95]. For the hyphal plug, *P. insidiosum* is grown on Sabouraud dextrose agar (SDA) at 37 °C for five days [69, 82]. An agar plug (5 mm in diameter) is excised from the edge of an actively-growing colony and used as inoculum in the radial growth method [69, 82, 87], the agar diffusion method [96, 97, 98, 99, 100, 101], or the broth microdilution method [100, 101, 102, 103, 104].

### 2.2 *In vitro* drug susceptibility methods

#### 2.2.1 Broth microdilution method

**Figure 1:**
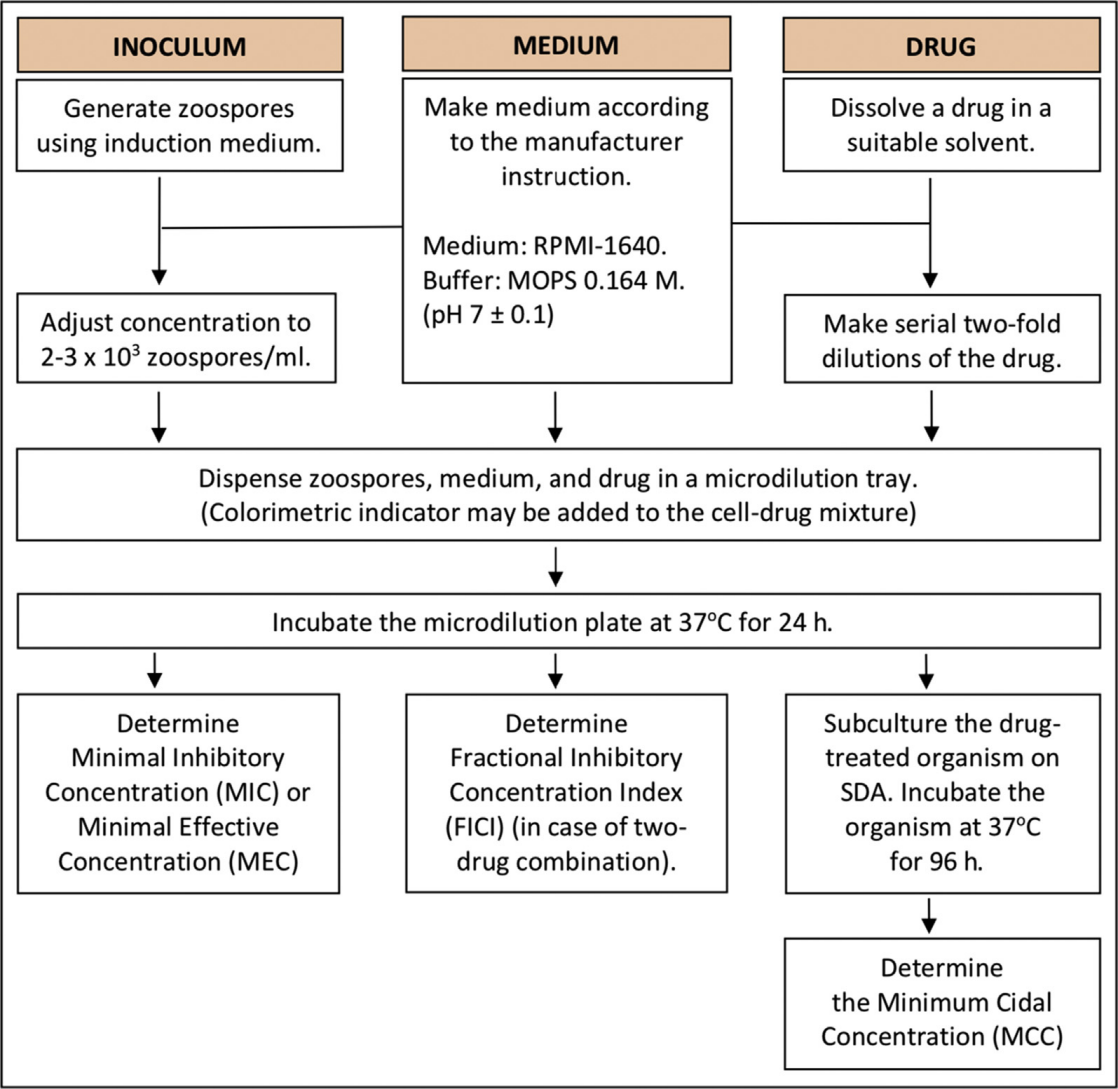
Broth microdilution method for susceptibility testing of an antimicrobial agent against *P. insidiosum*. The inoculum (zoospores), medium (RPMI-1640), and drug (in two-fold dilutions) are prepared for co-incubation in a microdilution plate. After incubation at 37 °C for 24 h, minimal inhibitory concentration (MIC), minimal effective concentration (MEC), fractional inhibitory concentration index (FICI), and minimal cidal concentration (MCC) can be determined.

By modifying the Clinical and Laboratory Standards Institute (CLSI) M38-A2 protocol, Pereira et al. used the broth microdilution method for susceptibility testing of *P. insidiosum* zoospores, as summarized in Figure 1 [81,105]. The generated zoospores are resuspended in RPMI-1640 broth, adjusted to pH 6.9–7.1 with 0.164 M 3-[N-morpholino] propane sulfonic acid (MOPS) and the final concentration of 2–3 x 10^3^ cells/ml [81, 106]. The zoospore suspension (inoculum) is tested against RPMI-1640 alone (no-drug control) and various drug concentrations in a microdilution tray. The tetrazolium salt (a colorimetric indicator) can be added in the drug-zoospore mixture to facilitate the assay interpretation (only the viable *P. insidiosum* hyphae turns purple) [106]. Minimal inhibitory concentration (MIC) or minimal effective concentration (MEC) is determined after 24-h incubation at 37 °C [81, 105, 106, 107]. MIC is the lowest drug concentration that visually demonstrates 100% growth inhibition [81, 106], whereas MEC is the lowest drug concentration that results in morphological changes of the organism [105, 107]. The minimum cidal concentration (MCC) is the lowest drug concentration that no growth is observed after incubating the drug-treated organism on a drug-free culture agar (i.e., SDA) for up to 96 h at 37 °C [79, 81, 90, 105].

Because the zoospore is challenging to generate in the laboratory, the hyphal agar plug can be alternatively used as the inoculum in the broth microdilution method [102, 103, 104]. The hyphal agar plugs are directly added into drug-containing broth. The susceptibility result is determined by weighing organism-dried weight [104], culturing drug-treated hyphae [100, 101, 103, 108], or directly observing organism growth by the naked eye [102].

To evaluate the effect of two-drug combination (i.e., drugs A and B), a fractional inhibitory concentration index (FICI) score is calculated, using the following formula: FICI = (MIC of drug A in the drug A-B combination/MIC of drug A) + (MIC of drug B in the drug A-B combination/MIC of drug B). The obtained FICI score defines synergistic (FICI ≤0.5), indifferent (0.5 < FICI ≤4), and antagonistic (FICI >4) interactions of the drug combination against *P. insidiosum* [107, 109].

#### 2.2.2 Radial growth method

Some investigators used the radial growth method to study drug susceptibility against *P. insidiosum* [69, 82, 87, 98, 102]. Briefly, a hyphal agar plug (as inoculum) is excised from the edge of an actively-growing *P. insidiosum* colony on SDA (or vegetable extract agar) and placed on a new agar plate containing various drug concentrations (including no-drug control) [69, 82, 87]. The hyphal side of the plug should be in direct contact with the drug-containing agar [82]. The agar plates are incubated at 37 °C for 2–3 days before measuring a colony diameter. The mean colony diameter of each strain is subtracted by the agar plug width (~5 mm) and divided by two to obtain the radial growth of drug-treated *P. insidiosum* [69, 82, 87, 102]. Radial growth-based MIC is the drug concentration that completely inhibits *P. insidiosum* growth.

#### 2.2.3 Agar diffusion method

**Figure 2:**
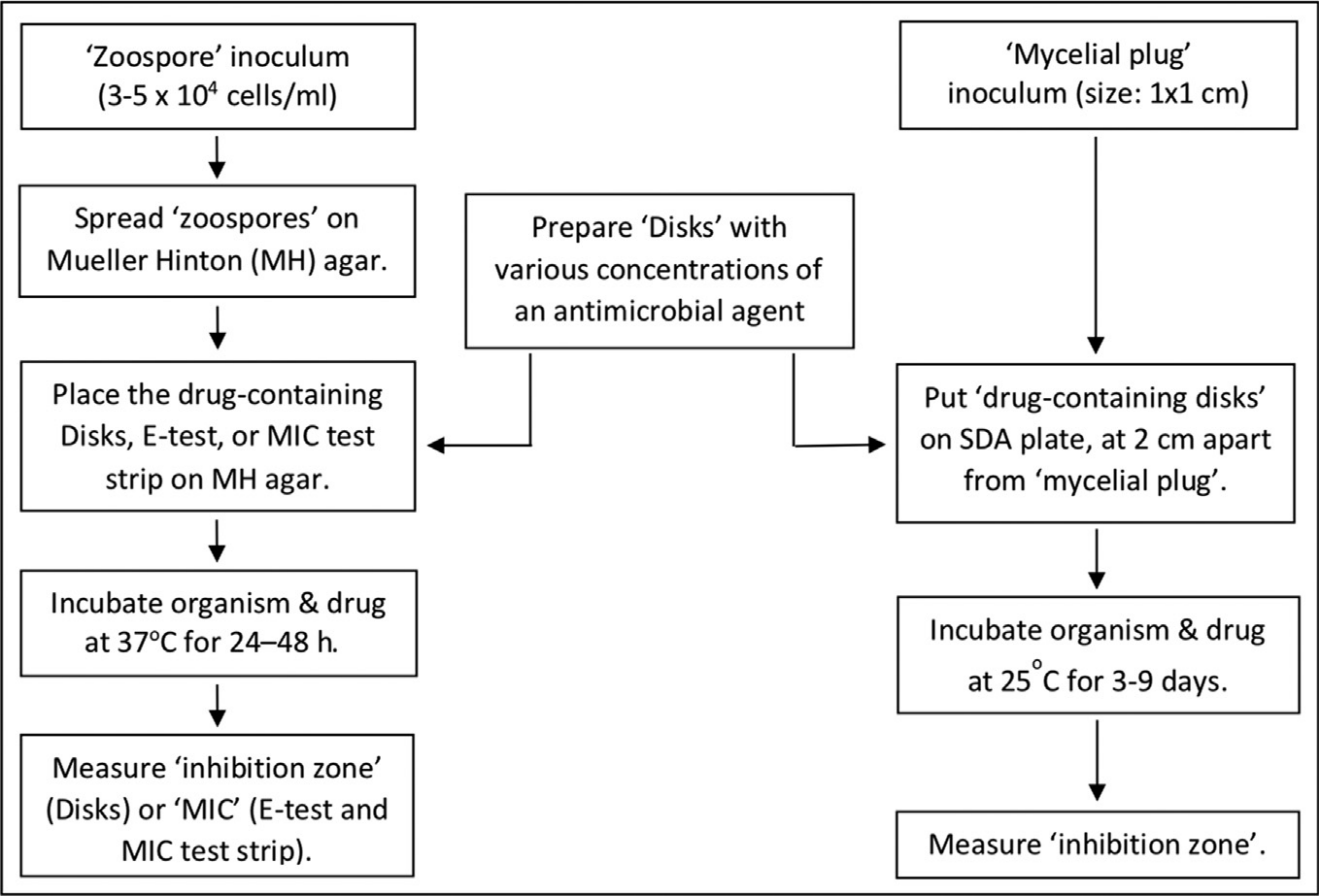
Agar diffusion method for susceptibility testing of an antimicrobial agent against *P. insidiosum*. Two types of inoculum can be used in this method: Zoospore suspension (which is spread on an agar plate) and Mycelial plug (which is placed 2-cm apart from a drug-containing disk on an agar plate). Disks with various concentrations of an antimicrobial drug are co-incubated with zoospores (at 37 °C for 24–48 h) or mycelial plugs (at 25 °C for 3–9 days). Antimicrobial effects of the drug can be determined as inhibition zone or minimal inhibitory concentration (MIC).

Zoospores [79, 88] and hyphal agar plugs [96, 97, 110, 111] have been used as inoculum in the agar diffusion susceptibility method, as depicted in Figure 2. The number of zoospores used in the agar diffusion method (3–5 x 10^4^ cells/ml) is markedly different from that used in the broth microdilution method (2–3 x 10^3^ cells/ml) [79, 81, 88, 106]. Approximately 200 μl of the zoospores suspension was spread on the entire surface of a non-supplemented Mueller Hinton (MH) agar plate, and the excess liquid is removed using a sterile pipette [88, 105]. A drug-containing disk is placed on the surface of each plate and incubated for 24–48 h at 37 °C before the measurement of the clear zone diameter [79, 88]. The commercial E-test (bioMérieux, France) or MIC Test Strip (Liofilchem, Italy) can replace the drug-containing disk, and MIC is read from the provided scale [79, 88].

Some natural compounds have been evaluated for their anti-*P. insidiosum* activities, using the agar diffusion method [96, 97, 98, 99, 100, 101, 110, 111]. A hyphal agar plug (1 × 1 cm in size) is placed in the center of an SDA plate and inoculated at room temperature. Afterward, a disk soaked with 20 μl of the natural compound or extract is put on the same SDA plate, placed 2 cm apart from the hyphal agar plug [112]. The inhibition zone is measured after prolonged incubation for 3–9 days at 25 °C.

### 2.3 *In vivo* drug susceptibility method

*In vivo* susceptibility study is clinically useful for the determination of drug efficacy against *P. insidiosum* [80, 81, 85, 103]. Rabbit is the commonly-used experimental model of pythiosis for *in vivo* drug susceptibility analysis [80, 86, 113, 114]. The animals are inoculated subcutaneously with ~2 × 10^4^ viable zoospores/ml, and *P. insidiosum* infection is usually established within 25 days post-inoculation [86, 113]. An antimicrobial agent is then administered in the infected animals [80, 81, 113, 114]. Changes in sizes of the lesion (i.e., subcutaneous nodular area), blood tests, microbiological workups (i.e., culture and PCR), and histopathologic results are used to assess the extent of *P. insidiosum* infection in response to the tested drug [80, 86, 113].

## 3 Antimicrobial agents against *P. insidiosum*

Several groups of antimicrobial drugs, such as antifungals, antibacterials, natural extracts, and some other compounds, have been investigated *in vitro* and *in vivo* for their anti-*P. insidiosum* effects, as summarized below:

### 3.1 Antifungal drugs

#### 3.1.1 Allylamines

Terbinafine was designed to inhibit the enzyme squalene epoxidase (ERG1) of the fungal sterol biosynthetic pathway [69]. It has been used in the treatment of pythiosis since its first report on the successful medical treatment of this disease [7, 51, 72, 115, 116, 117, 118, 119, 120]. However, administration of terbinafine, usually in combination with itraconazole, has shown a favorable response in only a few pythiosis patients [7, 72, 117, 120]. MICs of terbinafine varied (range: 0.5–128 μg/ml), depending on *P. insidiosum* strains tested (i.e., different genotypes) and the susceptibility methods used (i.e., broth dilution and radial growth) [69, 72, 86, 87, 88, 95, 107, 109, 117, 121, 122, 123, 124, 125, 126, 127, 132] (Table 1). Because *P. insidiosum* lacks the ERG1-encoding gene [69], it is still mysterious about how terbinafine exhibits antimicrobial activity against some strains of this pathogen.

**Table 1:** *In vitro* susceptibility testing of the conventional antifungal drugs against *P. insidiosum.*

Drug class	Drug name	Susceptibility method(s)	*P. insidiosum*		MIC (μg/ml)		Reference(s)
			Host (number of isolates)	Country of origin	Range	Mean	
Allylamines	Terbinafine	BMD	Horses (15–30)	Brazil	0.5–128	8.0–32.0	[86, 88, 95, 107, 109, 121, 122, 123, 124, 126]
		BMD	Humans (1–22)	Thailand	2–4	NA	[72, 117, 125, 132]
		RGM	Dogs (6)	USA	>8	>8	[87]
		RGM	Humans (30)	Thailand	>128	>128	[69]
Azoles	Miconazole	BMD	Horse (17–22)	Brazil	2–32	13.6	[95, 122]
	Ketoconazole	BMD	Horse (17–22)	Brazil	4–64	23.1	[95, 122]
	Itraconazole	BMD, ADM	Horse (15–30)	Brazil	≥16	>16	[86, 88, 95, 107, 121, 123, 126]
		BMD	Human (1–22)	Thailand	1–4	NA	[72, 117, 125, 132]
		RGM	Dog (6)	USA	>8	>8	[87]
		RGM	Human (30)	Thailand	>128	>128	[69]
	Voriconazole	BMD, ADM	Horse (28–30)	Brazil	≥16	>16	[88, 107, 121]
		BMD	Human (1–22)	Thailand	1–8	NA	[72, 117, 125, 132]
		RGM	Dog (6)	USA	>8	>8	[87]
	Fluconazole	BMD, ADM	Horse (17–28)	Brazil	≥32	59.0	[88, 122]
		BMD	Human (1–22)	Thailand	1–8	NA	[72, 117, 125, 132]
	Posaconazole	BMD, ADM	Horse (28)	Brazil	>32	>32	[88]
		RGM	Dog (6)	USA	>8	>8	[87]
Polyenes	Amphotericin B	BMD, ADM	Horse (17–30)	Brazil	4–128	25.1–34.3	[88, 107, 109, 123]
		BMD	Human (1–22)	Thailand	4–8	NA	[72, 117, 125, 132]
Echinocandins	Caspofungin	BMD, ADM	Horse (15–30)	Brazil	4–256	16.0–94.8	[81, 86, 88, 107, 122, 123, 137]
		BMD	Human (1–22)	Thailand	2–8	NA	[72, 117, 125, 132]
		RGM	Dog (6)	USA	>2	>2	[87]
	Anidulafungin	BMD, ADM	Horse (28–30)	Brazil	>32	1000.6	[88, 107]
		BMD	Human (1–22)	Thailand	2–8	NA	[72, 117, 125, 132]
	Micafungin	BMD, ADM	Horse (17–30)	Brazil	>32	776.0	[88, 107, 114]
Others	Griseofulvin	BMD	Human (1)	Thailand	>32	>32	[125]
	5-Fluorocytosine	BMD	Unknown (1)	China	4	4	[127]

**Abbreviations:** BMD, broth microdilution method; RGM, radial growth method; ADM, agar diffusion method; MIC, minimal inhibitory concentration; NA, data not available.

#### 3.1.2 Azoles

Azole drugs inhibit fungi by inactivating the 14-α-sterol demethylase (ERG11) [72,122,[128], [129], [130], [131]]. The ERG11-encoding gene is present in *P. insidiosum*, but phylogenetically diverse from that of the true fungi [69]. This finding suggests that *P. insidiosum* ERG11 may not be an optimal target of the azole drugs. The azoles comprise two subclasses: imidazoles (i.e., miconazole and ketoconazole) and triazoles (i.e., itraconazole, voriconazole, fluconazole, and posaconazole). These drugs had diverse *in vitro* antimicrobial activities against *P. insidiosum* (Table 1). For example, MICs of miconazole ranged from 2 to 32 μg/ml, whereas that of ketoconazole ranged between 4 and 64 μg/ml [95, 122]. Compared to imidazoles, triazoles generally exhibited a broader MIC range. MICs of itraconazole were reportedly different from study to study (Table 1), ranging from 1 to >128 μg/ml [69, 72, 86, 87, 88, 95, 107, 117, 121, 123, 125, 126, 132]. Voriconazole and fluconazole had MICs greater than 16 μg/ml [88, 107, 121] and 32 μg/ml [88, 122], respectively, against Brazilian isolates of *P. insidiosum*. In contrast, these two drugs inhibited Thai isolates at MICs lesser than 8 μg/ml [72, 117, 125, 132]. Posaconazole showed anti-*P. insidiosum* activity with MICs greater than 8 μg/ml [87, 88].

#### 3.1.3 Polyenes

The polyene drug, amphotericin B, binds ergosterol in the cell membrane and forms pores that lead to ion leakage and cell death [133]. MICs of amphotericin B, tested against the animal isolates of *P. insidiosum*, were 4–128 μg/ml [88, 107, 109, 123], while tested against the human strains, were 4–8 μg/ml [72, 117, 125, 132] (Table 1). The lack of the endogenous ergosterol (drug target), due to the incomplete ergosterol biosynthesis pathway in *P. insidiosum* [69], could explain clinical unresponsiveness to amphotericin B in some cases [8, 12, 71, 76, 116]. However, in the treatment of several horses with pythiosis, the intravenous regional limb perfusion of amphotericin B, in conjunction with surgical intervention and thermocautery, showed significant regression of the lesion, complete epithelialization, and no sign of recurrence during a one-year follow-up [134, 135].

#### 3.1.4 Echinocandins

Echinocandins (i.e., caspofungin, anidulafungin, and micafungin) were designed to inhibit β-1,3-D-glucan synthase, which forms glucan, a major cell wall component of fungi and oomycetes [31, 136]. Each echinocandin drug had a different anti-*P. insidiosum* activity (Table 1). When *P. insidiosum* isolates from horses in Brazil were tested by broth microdilution method, MICs of caspofungin ranged from 4 to 256 μg/ml [81, 86, 88, 107, 122, 123, 137], which were generally lower than MICs of anidulafungin (>32 μg/ml) [88, 107] and micafungin (>32 μg/ml) [88, 107, 114]. This observation suggests that caspofungin is more potent than the other echinocandin tested. As opposed to the horse strains, relatively-lower MICs of caspofungin and anidulafungin (2–8 μg/ml) were observed in the human isolates from Thailand [72, 117, 125, 132]. MEC (the lowest drug concentration that changes the microscopic morphology of the organism) has been used to evaluate the responsiveness of *P. insidiosum* to echinocandins. Caspofungin exhibited better MECs (8–32 μg/ml), compared to micafungin (16–128 μg/ml) and anidulafungin (≥256 μg/ml) [107, 114]. *In vivo* susceptibility of caspofungin against *P. insidiosum* in the rabbit model of pythiosis showed a reduced lesion size (i.e., subcutaneous nodule) and a decrease in hyphal burden [81, 113]. However, the subcutaneous lesion regrew when discontinuing caspofungin administration, indicating that the drug had, to some extent, a static microbial effect [81].

#### 3.1.5 Other antifungal drugs

Griseofulvin and 5-fluorocytosine, classified in two separated groups of antifungals, were also tested against *P. insidiosum* (Table 1). Regarding the mechanism of action, griseofulvin disrupts the microtubule function and the assembly of the mitotic spindle, while 5-fluorocytosine inhibits thymidylate synthetase and impairs DNA synthesis [131]. Griseofulvin had broth microdilution-based MIC of >32 μg/ml [125], whereas 5-fluorocytosine showed such MIC of 4 μg/ml [127] (Table 1).

### 3.2 Antibacterial drugs

Different classes of systemic and topical antibacterial drugs have been evaluated for their *in vitro* or *in vivo* anti-*P. insidiosum* activities. Compared with the antifungals, some antibacterial drugs exhibited a relatively-greater inhibitory activity against *P. insidiosum*. Recent reports on the susceptibility of *P. insidiosum* to the antibacterial drugs are summarized below and in Table 2.

**Table 2:** *In vitro* susceptibility testing of the conventional antibacterial drugs against *P. insidiosum.*

Drug class	Drug name	*P. insidiosum*		MIC (μg/ml)^a^		Inhibition zone (mm)^b^		References
		Host (number of isolates)	Country of origin	Range	Mean	Range	Mean	
Tetracyclines	Minocycline	Horse (25–30)	Brazil	0.06–4	0.2–1.0	21–40	31.9	[80, 88, 106, 107, 123]
		Horse (11)	USA, Costa Rica	0.25–4	1.1–2.0	NA	NA	[139]
		Human (38–48)	India	0.02–4	0.6	18–35	28.7	[9]
		Human (1)	Japan	NA	NA	Large inhibition zone		[141]
		Human (1–27)	Thailand	1–4	1.6–2.0	NA	NA	[125, 139]
		Environment (12)	Thailand	2–4	2.0–2.2	NA	NA	[139]
	Doxycycline	Horse (26–28)	Brazil	0.5–8	1.8–3.3	22–38	30	[88, 106]
		Horse (11)	USA, Costa Rica	1–16	3.4–4.0	NA	NA	[139]
		Human (38–48)	India	0.13–12	3.11	14–32	22.3	[9]
		Human (1–27)	Thailand	1–16	3.7–4.3	NA	NA	[125, 139]
		Environment (12)	Thailand	2–16	4.0–4.8	NA	NA	[139]
	Tetracycline	Horse (25–28)	Brazil	1–32	6.0–8.7	11–42	27.4	[88, 106, 140]
		Human (38–48)	India	0.19–24	5.09	16–34	23.7	[9]
	Oxytetracycline	Horse (26)	Brazil	2–32	7.4	NA	NA	[106]
Glycylcyclines	Tigecycline	Horse (24–30)	Brazil	0.03–4	0.2–1.3	23–40	32.2	[80, 88, 90, 107, 123]
		Horse (11)	USA, Costa Rica	0.5–2	1.1–2.0	NA	NA	[139]
		Human (38–48)	India	0.02–1.5	0.3	20–35	27.2	[9]
		Human (27)	Thailand	1–4	1.2–1.6	NA	NA	[139]
		Environment (12)	Thailand	2–4	2.0–2.2	NA	NA	[139]
Macrolides	Clarithromycin	Horse (25–30)	Brazil	0.25–64	1.4–4.5	20–38	28.3	[79, 80, 88, 106, 107, 123]
		Horse (11)	USA, Costa Rica	0.13–2	1.0–1.4	NA	NA	[139]
		Human (38–48)	India	0.05–4	1.7	6–34	20.5	[9]
		Human (1–27)	Thailand	0.13–8	0.5–1.7	NA	NA	[125, 139]
		Environment (12)	Thailand	1–4	1.8–2.0	NA	NA	[139]
	Azithromycin	Horse (21–30)	Brazil	0.03–32	0.7–6.9	14–40	29.2	[79, 80, 88, 106, 107, 123, 124]
		Horse (11)	USA, Costa Rica	2–8	2.7–2.8	NA	NA	[139]
		Human (38–48)	India	0.02–32	5.4	6–33	22.1	[9]
		Human (1)	Japan	NA	NA	Intermediate inhibition zone		[141]
		Human (1–27)	Thailand	1–16	3.1–5.3	NA	NA	[125, 139]
		Environment (12)	Thailand	2–16	4.0–4.8	NA	NA	[139]
	Erythromycin	Horse (25–28)	Brazil	1–32	6.4–7.7	0–34	22.9	[88, 106, 140]
		Human (1)	Japan	NA	NA	Intermediate inhibition zone		[141]
	Roxithromycin	Horse (28)	Brazil	2–128	9.7	10–34	18.9	[88]
	Josamycin	Horse (30)	Brazil	2–64	16	NA	NA	[79]
	Tilmicosin	Horse (28)	Brazil	4–128	27.6	0–28	17.6	[88]
Pleuromutilins	Retapamulin	Horse (30)	Brazil	0.25–32	1.45	NA	NA	[79]
	Valnemulin	Horse (30)	Brazil	0.25–16	2.09	NA	NA	[79]
	Tiamulin	Horse (30)	Brazil	2–64	16.4	NA	NA	[79]
Streptogramins	Quinupristin and Dalfopristin	Horse (25–28)	Brazil	0.5- >32	2.8–5.8	NA	NA	[88, 140]
Lincosamides	Clindamycin	Horse (25–28)	Brazil	2- >256	7.0–16.0	0–21	11.5	[88, 140]
	Lincomycin	Horse (28)	Brazil	>256	>256	No inhibition zone		[88]
Oxazolidinones	Linezolid	Horse (25–30)	Brazil	0.5–64	1.7–13.3	18–46	31.5	[79, 88, 140]
		Horse (11)	USA, Costa Rica	4–8	5.4–8.0	NA	NA	[139]
		Human (38–48)	India	0.75–32	7.7	20–44	31.2	[9]
		Human (1)	Japan	NA	NA	Large inhibition zone		[141]
		Human (1–27)	Thailand	4–32	8.0–9.2	NA	NA	[125, 139]
		Environment (12)	Thailand	4–16	9.5	NA	NA	[139]
	Sutezolid	Horse (30)	Brazil	4–64	7.5	NA	NA	[79]
	Tedizolid	Horse (30)	Brazil	>32	>32	NA	NA	[79]
Phenicols	Florfenicol	Horse (28)	Brazil	8- >256	25.1	0–39	28.6	[88]
	Chloramphenicol	Horse (25–28)	Brazil	2- >256	23.1–27.1	0–40	26.3	[88, 140]
		Human (38–48)	India	16–256	204.6	6–25	12.2	[9]
		Human (1)	Japan	NA	NA	Intermediate inhibition zone		[141]
Aminoglycosides	Paromomycin	Horse (24)	Brazil	32–64	49.3	NA	NA	[90]
	Streptomycin	Horse (24–28)	Brazil	32–64	50.7	No inhibition zone		[88, 90]
		Horse (11)	USA, Costa Rica	16- >32	26.9- >32	NA	NA	[139]
		Human (1–27)	Thailand	16- >32	22.6- >32	NA	NA	[125, 139]
		Environment (12)	Thailand	>32	>32	NA	NA	[139]
	Gentamicin	Horse (24–28)	Brazil	>8	55.3	No inhibition zone		[88, 90, 140]
		Horse (11)	USA, Costa Rica	16- >32	26.9- >32	NA	NA	[139]
		Human (1–27)	Thailand	>32	>32	NA	NA	[125, 139]
		Environment (12)	Thailand	>32	>32	NA	NA	[139]
	Neomycin	Horse (24–28)	Brazil	32–64	55.5	No inhibition zone		[88, 90]
		Horse (11)	USA, Costa Rica	32- >32	32- >32	NA	NA	[139]
		Human (1–27)	Thailand	32- >32	32- >32	NA	NA	[125, 139]
		Environment (12)	Thailand	>32	>32	NA	NA	[139]
	Tobramycin	Horse (25–28)	Brazil	>8	>8	No inhibition zone		[88, 140]
		Horse (11)	USA, Costa Rica	>32	>32	NA	NA	[139]
		Human (1)	Japan	NA	NA	No inhibition zone		[141]
		Human (1–27)	Thailand	>32	>32	NA	NA	[125, 139]
		Environment (12)	Thailand	>32	>32	NA	NA	[139]
	Kanamycin	Horse (25)	Brazil	>4	>4	NA	NA	[140]
	Amikacin	Horse (25)	Brazil	>32	>32	NA	NA	[140]
		Horse (11)	USA, Costa Rica	>32	>32	NA	NA	[139]
		Human (1–27)	Thailand	>32	>32	NA	NA	[125, 139]
		Environment (12)	Thailand	>32	>32	NA	NA	[139]
Others	Rifampicin	Horse (17–25)	Brazil	>2	61.4	NA	NA	[109, 140]
	Metronidazole	Horse (17)	Brazil	32–128	66.6	NA	NA	[109]
	Nitrofurantoin	Horse (25)	Brazil	64- >64	105.4	NA	NA	[140]

Abbreviations: MIC, minimal inhibitory concentration; NA, data not available.

a Minimal inhibitory concentration measured by broth microdilution method and agar diffusion method (E-test and MIC test strip).

b Inhibition zone measured by agar diffusion method (Disk diffusion).

#### 3.2.1 Tetracyclines and glycylcyclines

Tetracyclines inhibit protein synthesis by binding the 30S bacterial ribosomal and blocking the access of aminoacyl tRNA to the mRNA-ribosome complex [138]. Based on the broth microdilution method, minocycline, doxycycline, tetracycline, and oxytetracycline can differently suppress *P. insidiosum* growth at MICs of 0.02–4 μg/ml [9, 80, 88, 106, 107, 123, 125, 139], 0.13–16 μg/ml [9, 88, 106, 125, 139], 0.19–32 μg/ml [9, 88, 106, 140], and 2–32 μg/ml [106], respectively (Table 2). Based on the agar diffusion method, the mean inhibition zones of *P. insidiosum* were not markedly different: 28.7–31.9 mm for minocycline, 22.3–30 mm for doxycycline, and 23.7–27.4 mm for tetracycline [9, 88, 141] (Table 2). *In vivo* susceptibility information of minocycline showed a 17% cure rate in the rabbit model of pythiosis [80].

Glycylcyclines are synthetic analogs of the tetracyclines and share the mechanism of action [142]. Tigecycline, a derivative of minocycline, had MICs of 0.02–4 μg/ml [9, 80, 88, 90, 107, 123, 139] and the mean inhibition zones of 27.2–32.2 mm [9, 88] against *P. insidiosum* (Table 2). *In vivo* evaluation of tigecycline showed an increased lung invasion of *P. insidiosum* in one out of six experimental rabbits with pythiosis [80].

#### 3.2.2 Macrolides

Macrolides suppress the peptidyl transferase or block the ribosome exit tunnel of a nascent peptide [143]. Broth microdilution-based MIC ranges of the macrolide drugs against *P. insidiosum* have been reported (Table 2): clarithromycin, 0.05–64 μg/ml; azithromycin, 0.02–32 μg/ml; erythromycin, 1–32 μg/ml; roxithromycin, 2–128 μg/ml; josamycin, 2–64 μg/ml; and tilmicosin, 4–128 μg/ml [9, 79, 80, 88, 106, 107, 123, 124, 125, 139, 140]. The agar diffusion method with macrolides demonstrated different inhibition zones of *P. insidiosum* (Table 2): azithromycin (22.1–29.2 mm), clarithromycin (20.5–28.3 mm), erythromycin (22.9 mm), roxithromycin (18.9 mm), and tilmicosin (17.6 mm) [9, 88, 141]. *In vivo* susceptibility study in the rabbit model of pythiosis revealed an increased *P. insidiosum* burden for clarithromycin and an 83% cure rate for azithromycin [80].

#### 3.2.3 Pleuromutilins, streptogramins, and lincosamides

The antibacterial mechanism of pleuromutilins, streptogramins, and lincosamides are similar to that of macrolides. They inhibit peptidyl transferase in the large ribosomal subunit or interfere with polypeptide elongation [143, 144, 145, 146]. The broth microdilution-based MICs of these drug classes against *P. insidiosum* were summarized in Table 2. MIC ranges of the pleuromutilins were 0.25–32 μg/ml for retapamulin, 0.25–16 μg/ml for valnemulin, and 2–64 μg/ml for tiamulin [79]. MICs of the combination of quinupristin and dalfopristin ranged from 0.5 to >32 μg/ml [88, 140]. MICs of the lincosamides were from 2 to >256 μg/ml for clindamycin [88, 140] and >256 μg/ml for lincomycin [88]. Information on the mean inhibition zones of *P. insidiosum* (based on agar diffusion method) was available for two drugs: clindamycin (11.5 mm) and lincomycin (no inhibition zone) [88].

#### 3.2.4 Oxazolidinones

Oxazolidinones bind P site of the 50S ribosomal subunit and prevent the formation of a large ribosomal-fMet-tRNA complex that initiates protein synthesis [145]. The oxazolidinones drugs can suppress the growths of *P. insidiosum* at MICs of 0.5–64 μg/ml for linezolid, 4–64 μg/ml for sutezolid, and >32 μg/ml for tedizolid [9, 79, 88, 125, 139, 140] (Table 2). Linezolid showed the mean inhibition zone of 31.2–31.5 mm [9, 88, 141] (Table 2).

#### 3.2.5 Phenicols

Phenicols (also known as amphenicols) prevent the binding of the aminoacyl tRNA to the 50S bacterial ribosomal subunit and inhibit protein synthesis [145, 146, 147]. By the broth microdilution method, MICs of florfenicol and chloramphenicol, against *P. insidiosum*, were in the range of 2 to >256 μg/ml [9, 88, 140]. The agar diffusion method, using these two drugs, showed the mean inhibition zones in the range of 12.2–28.6 mm [9, 88, 141] (Table 2).

#### 3.2.6 Aminoglycosides

Aminoglycosides bind polysomes and interfere with protein synthesis by causing misreading and premature termination of mRNA translation [145]. In general, aminoglycosides, such as paromomycin, streptomycin, gentamicin, neomycin, tobramycin, kanamycin, and amikacin, had anti-*P. insidiosum* effect at broth microdilution-based MICs of >4 μg/ml (Table 2) [88, 90, 125, 139, 140]. Some investigators demonstrated that these drugs could exhibit MICs of up to 64 μg/ml [90]. Besides, half of *P. insidiosum* isolates tested had a significant reduction in dried weight after exposed to streptomycin [104]. Based on the agar diffusion method, aminoglycosides showed no inhibition zone of *P. insidiosum* [88, 141].

#### 3.2.7 Other antibacterial drugs

Rifampicin, metronidazole, and nitrofurantoin have been evaluated for anti-*P. insidiosum* activities, using the broth microdilution method (Table 2). MICs of these drugs were diverse: for example, >2 μg/ml for rifampicin, from 32 to 128 μg/ml for metronidazole, and from 64 to >64 μg/ml for nitrofurantoin [109, 140]. *P. insidiosum* has been tested against some other drugs, such as fusidic acid (MIC >256 μg/ml), daptomycin (>4 μg/ml), novobiocin (>1.6 μg/ml), optochin (concentration not defined), quinolones (>4 μg/ml), vancomycin (>16 μg/ml), bacitracin (concentration not defined), trimethoprim+sulfamethoxazole (>2+38 μg/ml), polymyxins (≥8 μg/ml), carbapenems (>4 μg/ml), penicillins (>8 μg/ml), and cephalosporins (>2 μg/ml) [88, 125, 139, 140, 141].

#### 3.2.8 Topical antimicrobial drugs

Several topical antiseptics showed antimicrobial activities against *P. insidiosum*. Most of the topical antimicrobials tested (i.e., triclosan, mupirocin, cetylpyridinium chloride, benzalkonium chloride, and cetrimide) had MICs less than 32 μg/ml [9, 88, 124, 140]. Crystal violet completely inhibited the growths of all *P. insidiosum* isolates studied [140]. No anti-*P. insidiosum* activity was observed with potassium permanganate at the maximal concentration tested (64 μg/ml) [124].

### 3.3 Natural extracts

#### 3.3.1 Plant-extracted essential oils

Plant-extracted essential oils from *Origanum vulgare*, *Origanum majorana*, *Mentha piperita*, *Rosmarinus officinalis*, and *Melaleuca alternifolia* have shown *in vitro* antimicrobial effect against *P. insidiosum* (Table 3). For example, *O. vulgare*-derived oil mainly consisted of carvacrol (71–93%), possessed MICs of 50–1,750 μg/ml [94, 126, 148], and the purified carvacrol had MICs of 80–320 μg/ml [123]. The extracted oils from *O. majorana* (containing 34% of 4-terpineol), *M. piperita* (30–58% of menthone) and *R. officinalis* (65% of 1,8-cineole) demonstrated MICs of 50–3,500 μg/ml, 110–3,500 μg/ml, and 110–3,500 μg/ml, respectively [94, 126, 148]. *M. alternifolia* oil (containing 40–52% of terpinene-4-ol) exhibited MICs of 531–2,125 μg/ml [94, 126, 149]. Similarly, nanoemulsion (mixed with 1% of *M. alternifolia* oil) showed MICs of 133–2,125 μg/ml [149].

**Table 3:** *In vitro* susceptibility testing of the natural compounds against *P. insidiosum*.

Source of compound	Identified compound(s)	*P. insidiosum*		MIC (μg/ml)^a^	Inhibition zone (mm)^b^ (concentration)	Reference(s)
		Host (number of isolates)	Country of origin			
*Origanum vulgare* oil	Carvacrol	Horse (20–22)	Brazil	50-1,750	NA	[94, 126, 148]
Purified carvacrol	Carvacrol	Horse (25)	Brazil	80–320	NA	[123]
*Origanum majorana* oil	4-terpineol	Horse (22)	Brazil	50-3,500	NA	[148]
*Mentha piperita* oil	Menthone	Horse (20–22)	Brazil	110-3,500	NA	[94, 126, 148]
*Rosmarinus officinalis* oil	1,8-cineole	Horse (22)	Brazil	110-3,500	NA	[148]
*Melaleuca alternifolia* oil	Terpinene-4-ol	Horse (20–26)	Brazil	133-2,125	NA	[94, 126, 149]
*Micromelum falcatum* (fruit)^c^	Isomicromelin	Unknown (1)	Thailand	NA	21.0 (0.22 mM)	[97]
	Micromarin B	Unknown (1)	Thailand	NA	19.2 (0.21 mM)	[97]
	7-methoxy-8-(4′-methyl-3′-furanyl)coumarin	Unknown (1)	Thailand	NA	15.5 (0.20 mM)	[97]
	Secomicromelin	Unknown (1)	Thailand	NA	6.2 (0.22mM)	[97]
*Alyxia schlechteri* (root)^c^	Pinoresinol	Unknown (1)	Thailand	NA	16.1 (76 μg/μl)	[110]
	Alyterinate C	Unknown (1)	Thailand	NA	16.0 (73 μg/μl)	[110]
	Medioresinol	Unknown (1)	Thailand	NA	13.3 (65 μg/μl)	[110]
*Clausena harmandiana* (root)^c^	Clausine K	Unknown (1)	Thailand	NA	16.2 (10 μg/μl)	[111]
	Zapoterin	Unknown (1)	Thailand	NA	11.8 (40 μg/μl)	[111]
	Clausine L	Unknown (1)	Thailand	NA	10.2 (40 μg/μl)	[111]
	N-methylswietenidine B	Unknown (1)	Thailand	NA	7.9 (58 μg/μl)	[111]
*Dalbergia stipulacea* (stem)^c^	(-)-vestitol	Human (1)	Thailand	NA	2.9–9.8 (1-1,000 μg/ml)	[99]
	2′,4′,4′-trihydroxy chalcone	Human (1)	Thailand	NA	3.8–5.1 (10–1,000 μg/ml)	[99]
	Dihydromaackiain	Human (1)	Thailand	NA	7.4–7.7 (100–1,000 μg/ml)	[99]
	Mucronulatol	Human (1)	Thailand	NA	5.9–6.6 (100–1,000 μg/ml)	[99]
	Dalpulanone	Human (1)	Thailand	NA	4.9–6.7 (100–1,000 μg/ml)	[99]
	Duartin	Human (1)	Thailand	NA	3.7–4.2 (100–1,000 μg/ml)	[99]
*Stryphnodendron adstringens* (bark)^d^	Tannin	Horse (15)	Brazil	1,000–1,500	NA	[103]
Purified tannin	Tannin	Horse (15)	Brazil	500–1000	NA	[103]
*Allium sativum*^e^	Allicin	Horse (17)	Brazil	<6,250	NA	[150]
Africanized honeybees propolis^f^	Benzoic acid, coumaric acid, caffeic acid, artepillin C, etc.	Horse (15)	Brazil	3.4	NA	[108]
*Melipona fasciculata* geopropolis^f^	Triterpenes, anacardic acid, alkylresorcinols, etc.	Horse (15)	Brazil	12.5	NA	[108]
*Pseudomonas stutzeri* ST1302^g^	Fraction number 6	Unknown (11)	Thailand	3.13	NA	[100, 101]
*Klebsiella pneumoniae* ST2501^g^	Fraction number 1	Unknown (11)	Thailand	1.57–3.13	NA	[100, 101]

Abbreviations: MIC, minimal inhibitory concentration; NA, data not available.

a Minimal inhibitory concentration measured by broth microdilution method.

b Inhibition zone measured by agar diffusion method (Disk diffusion).

c Extraction using ethyl acetate and methanol.

d Extraction using methanol.

e Extraction using alcohol.

f Extraction using ethanol.

g Metabolites.

A combination of *M. piperita* and *O. vulgare* oils had synergized antimicrobial effects against 65% of *P. insidiosum* isolates tested [94]. However, *M. alternifolia* oil, combined with either *M. piperita* or *O. vulgare* oil, showed no additional anti-*P. insidiosum* activity [94]. A mixture of the antifungal drug itraconazole (but not terbinafine) and either *M. alternifolia*, *M. piperita*, or *O. vulgare* oil increased the inhibitory effect on 60–95% of the recruited *P. insidiosum* isolates [126]. When *O. vulgare* and *M. piperita* oil were topically applied, in conjunction with *P. insidiosum* antigen administration (so-called immunotherapy), the skin lesion in the rabbit model of pythiosis was relatively smaller, compared with applying each oil alone [83].

#### 3.3.2 Plant-extracted compounds

Some compounds extracted from the plants using ethyl acetate and methanol can suppress *P. insidiosum* growths (Table 3). For instance, isomicromelin, micromarin B, 7-methoxy-8-(4′-methyl-3′-furanyl)coumarin, and secomicromelin were derived from *Micromelum falcatum* fruit and at the concentration of 0.20–0.22 mM, showed *P. insidiosum*-inhibited zones of 6.2–21 mm [97]. *Alyxia schlechteri* root-extracted pinoresinol, alyterinate C, and medioresinol (at the concentration of 65–76 μg/μl) affected *P. insidiosum* growths by showing the inhibition zones of 13.3–16.1 mm [110]. Likewise, the clausine K, zapoterin, clausine L, and N-methylswietenidine B (extracted from *Clausena harmandiana* root; at the concentration of 10–58 μg/μl) gave the inhibition zones of 7.9–16.2 mm [111]. MICs of xanthyletin and 4-formylsyringol (crude extracts of *Scaevola taccada* fruit) were 5 and 10 μg/ml, respectively [96]. Compared with the purified xanthyletin of *S. taccada*, the synthetic version of this compound showed lower MIC (3 vs. 5 μg/ml) or greater antimicrobial effect against *P. insidiosum* [96, 100, 101], and it was not toxic to fibroblast cell lines [100]. The vestilol, trihydroxy chalcone, dihydromaackiain, mucronulatol, dalpulanone, and duartin extracted from *Dalbergia stipulacea* stem (concentration: 1,000 μg/ml) showed 9.8, 5.1, 7.7, 6.6, 6.7, and 4.2 mm inhibition zones against *P. insidiosum*, respectively [99].

The aqueous phase alcohol extract of the garlic *Allium sativum* (mainly composed of allicin) had the anti-*P. insidiosum* MIC of <6,250 μg/ml [150] (Table 3). The methanol extract of *Stryphnodendron adstringens* bark (containing 46% of tannin) showed the minimal cidal concentrations (MCC) of 1,000–1,500 μg/ml against *P. insidiosum* growth, while the purified tannin possessed lower MCCs (<1,000 μg/ml) [103] (Table 3). The scanning electron microscopy demonstrated an altered cell wall of the tannin-treated *P. insidiosum* [103]. Nevertheless, either extracted or commercial tannin failed to recover the experimental rabbits with pythiosis [103].

#### 3.3.3 Other natural compounds

Bees produce propolis and geopropolis that exhibit antimicrobial activities [108]. These natural substances were ethanol extracted from the selected bees and used to explore the anti-*P. insidiosum* effect [108]. The extracted propolis (from Africanized honeybees) and geopropolis (from *Melipona* stingless bee) had MCCs of 3.4 and 12.5 mg/ml, respectively (Table 3). Approximately 10 μl of synthetic volatile organic compounds of the endophytic fungus *Muscodor crispans* (strain B23) can completely suppress the growths of all *P. insidiosum* isolates tested [82]. Some bacterial metabolites were reportedly active against *P. insidiosum*. For example, diketopiperazine and pyrrolnitrine of *Pseudomonas stutzeri* (strain ST1302) can inhibit the pathogen [98]. Besides, the metabolite of *Klebsiella pneumoniae* (strain ST2501) had a relatively-stronger anti-*P. insidiosum* activity than that of *P. stutzeri* [100, 101] (Table 3).

### 3.4 Other anti-*P. insidiosum* substances

The other substances that are not grouped with the drugs mentioned above were evaluated for the inhibition of *P. insidiosum* growths. For example, biogenic silver nanoparticles had an anti-*P. insidiosum* MIC range of 0.06–0.47 μg/ml [84]. The effect of the biogenic silver nanoparticle included the destruction of the cell wall and intracellular organelles. The cytotoxic concentration of the nanoparticle was twice as much compared with its effective concentration. Diphenyl diselenide showed MICs of 0.5–2 μg/ml, and this organoselenium compound temporarily reduced the lesion size in the rabbit model of pythiosis [85]. The agricultural fungicide mefenoxam (at 1 μg/ml) can completely inhibit 90% of *P. insidiosum* isolates tested [87]. Miltefosine is an alkylphosphocholine drug that possesses potent antiparasitic and antimicrobial activities, and it can inhibit *P. insidiosum* at MICs of 0.5–64 μg/ml [79, 151]. However, miltefosine showed a favorable response in the rabbit model of pythiosis [151]. Copper acetate and cadmium acetate are metal compounds that exhibited anti-*P. insidiosum* activity with MICs of 4–64 and 16–256 μg/ml, respectively [152].

Drug repurposing is a strategy to use a drug designed for one particular disease in another condition [153, 154]. Such a strategy has been applied to identify some drugs with anti-*P. insidiosum* effect. For instance, disulfiram, designed for the treatment of alcoholism, showed broth microdilution-based MICs of 8–32 μg/ml [102]. Deferasirox is an iron-chelating drug that had anti-*P. insidiosum* property with MICs of 12.5–50 μg/ml [114, 155]. Although deferasirox destroyed the hyphae and minimized the lesion size, it seemed to promote the dissemination of *P. insidiosum* infection [155, 156]. The lipid-controlling drug, fluvastatin, provided the anti-*P. insidiosum* MIC of >16 μg/ml [86, 109]. Ibuprofen, a nonsteroidal anti-inflammatory drug, showed anti-*P. insidiosum* activity with a broad MIC range of 128–2,048 μg/ml [86, 109].

### 3.5 Drug combinations

A combination of different antimicrobial drugs could contribute to a synergistic, indifferent, or antagonistic effect on *P. insidiosum* growth. Such an effect can be determined by using the MIC-based checkerboard technique [157]. The combination of two antifungal drugs, such as terbinafine and either amphotericin B, itraconazole, fluconazole, voriconazole, ketoconazole, miconazole, or caspofungin, resulted in an indifferent anti-*P. insidiosum* activity in 53–100% of the recruited isolates [72, 86, 109, 117, 121, 122, 127, 132]. Combinations of antibacterial drugs from different classes (i.e., glycylcyclines, tetracyclines and macrolides) were analyzed for anti-*P. insidiosum* effects *in vitro* [80, 125, 139]. Several combinations showed a favorable susceptibility outcome. For example, minocycline, combined with either tigecycline, azithromycin or clarithromycin had markedly synergistic anti-*P. insidiosum* effects in ~80% of the *P. insidiosum* isolates tested [80, 139]. However, such drug combinations had an anti-*P. insidiosum* effect in only 17% (minocycline and clarithromycin), 33% (minocycline and tigecycline), and 67% (minocycline and azithromycin) of the experimental rabbits with pythiosis [80].

The effects of antifungal-antibacterial drug combinations on *in vitro* growths of *P. insidiosum* were also investigated, as summarized in Table 4 [107,109]. All pairs of the selected antifungal and antibacterial drugs resulted in indifference in 27–94% of the isolates tested. Only a few sets of combined drugs (i.e., itraconazole and minocycline; micafungin and tigecycline or clarithromycin) provided a synergistic effect in ~70% of the analyzed isolates. To a lesser extent, several drug combinations (i.e., itraconazole and clarithromycin; terbinafine and rifampicin) exhibited antagonistic activity in up to 7% of the isolates. In two Thai patients with relapsed or inoperable vascular pythiosis, a combination of an antifungal drug (itraconazole or voriconazole) and a few antibacterial agents (i.e., doxycycline, azithromycin, or clarithromycin) can suppress the disease progression during the 64-week follow-up [125]. Drug selection and combination reported in these patients were guided by the susceptibility data [125].

**Table 4:** *In vitro* susceptibility testing of the combinations of antifungal (i.e., terbinafine, amphotericin B, itraconazole, voriconazole, caspofungin, anidulafungin, and micafungin) and antibacterial (i.e., minocycline, tigecycline, azithromycin, clarithromycin, metronidazole, rifampicin) drugs against *P. insidiosum.*

Drugs	Ratio (%) of Synergism: Indifference: Antagonism of each drug combination (antimicrobial activities were measured by broth microdilution)						
	Terbinafine	Amphotericin B	Itraconazole	Voriconazole	Caspofungin	Anidulafungin	Micafungin
Minocycline	67:33:00	73:27:00	70:30:00	60:40:00	46:47:07	43:57:00	63:37:00
Tigecycline	60:40:00	57:43:00	47:53:00	40:60:00	47:53:00	43:53:04	73:27:00
Azithromycin	33:67:00	40:57:03	30:67:03	53:47:00	43:53:04	43:53:04	67:33:00
Clarithromycin	63:37:00	63:37:00	43:50:07	57:43:00	53:43:03	47:50:03	70:30:00
Metronidazole	06:94:00	NA:94:NA	NA	NA	NA	NA	NA
Rifampicin	00:94:06	NA	NA	NA	NA	NA	NA

- Data were summarized from [107, 109].

- Combinations of metronidazole (or rifampicin) and other drugs were tested against 17 isolates, whereas the other drug combinations were tested against 30 isolates.

- Susceptibility interpretation of Echinocandins (i.e., caspofungin, anidulafungin, and micafungin) was based on Minimal Effective Concentration (MEC).

- Abbreviation: NA, data not available.

Drug combinations of either terbinafine or azithromycin and a topical antimicrobial agent (i.e., potassium permanganate, cetylpiridinium, triclosan, mupirocin, and benzalkonium) showed indifferent anti-*P. insidiosum* activity in at least 60% of the strains tested [124]. However, drug synergism can be observed in 71% of the analyzed *P. insidiosum* isolates, if terbinafine was combined with the topical drug cetrimide [124]. When combined with itraconazole, clarithromycin, azithromycin, minocycline, or tigecycline, either carvacrol or thymol (found in plant-extracted oil) had a synergistic outcome in most (60–96%) of the studied isolates [123].

Combinations of antimicrobial and repurposed drugs have shown additionally anti-*P. insidiosum* activities *in vitro* [86, 109, 114, 123]. Micafungin, combined with deferasirox, showed a synergistic effect in 88% of the tested isolates [114]. A three-drug combination of terbinafine, itraconazole, caspofungin, fluvastatin, and ibuprofen demonstrated an indifferent antimicrobial activity in 53–86% of the isolates [86]. The terbinafine-itraconazole-fluvastatin combination showed decreased hyphae burden in the rabbits with pythiosis [86]. However, prominent antagonistic drug interaction was observed in 35% of *P. insidiosum* isolates when the terbinafine-fluvastatin combination was tested [109]. Caution should be raised when using a certain drug combination *in vivo*, such as terbinafine and caspofungin [86], and micafungin and deferasirox [114], since such combinations might promote disseminated pythiosis, seen in the rabbit model.

## 4 Prospective and conclusion

The management of pythiosis is challenging, and in most cases, relies on combined treatment modalities: antimicrobial drugs, surgical intervention, and immunotherapy [7, 8, 49]. While radical surgery could aim at a cure of pythiosis, it leads to disabilities. In some humans and animals with advanced disease, surgical intervention is impossible or provides an unfavorable outcome. The efficacy of the immunotherapy alone, particularly in human patients with pythiosis, has not been evaluated clearly [7, 71, 72, 73, 118, 119]. A handful of conventional antifungal and antibacterial drugs possessed a prominent *in vitro* anti-*P. insidiosum* effect (Tables 1 and 2). Some antifungal and antibacterial drugs can decrease *P. insidiosum* burden and increase the survival rate in the animal model [80, 81, 89, 113]. The synergized anti-*P. insidiosum* effect has been observed when several drugs were combined [80, 86, 125, 139]. The use of some drugs, such as tigecycline [80], clarithromycin [80] and deferasirox [114, 155], could increase *P. insidiosum* burden and promote disseminated infection in the experimental rabbits. These possible outcomes should be considered when using such drugs clinically against *P. insidiosum*.

Drug selection and combination could be guided by *in vitro* susceptibility testing against the patient isolate of *P. insidiosum*. For example, co-administration of itraconazole and terbinafine showed the best *in vitro* anti-*P. insidiosum* effect, and significantly improved the condition of an American patient with invasive pythiosis without surgical intervention [120]. Two Thai vascular pythiosis patients with the inoperable disease can be controlled, during a long follow-up period (over a year), by administrating several antifungal and antibacterial drugs [125]. Besides, there are reports of the successful medical treatment in two Indian and Japanese patients with ocular pythiosis, using the combination of the topical and oral antimicrobial drugs [141, 158, 159]. Some dogs survived intestinal pythiosis after the treatment with corticosteroid and a terbinafine-itraconazole combination, without surgery [160]. These success stories on the management of pythiosis emphasize the clinical usefulness of the *in vitro* and *in vivo* susceptibility data.

The standard CLSI guideline is not available for *in vitro* drug susceptibility testing against *P. insidiosum*. Several in-house susceptibility methods (including broth microdilution method, radial growth method, and agar diffusion method) have been introduced to feasibly assess anti-*P. insidiosum* effect of various drugs and substances. Inoculum can be prepared from the zoospores or hyphae of *P. insidiosum*. Selection of a suitable susceptibility method and inoculum type depends on the nature of the substance used, availability of required reagents, skilled personal and objective of the experiment. Interpretation of *in vitro* susceptibility results (i.e., MIC, inhibition zone) needs to be evaluated clinically to establish a guideline on drug selection and combination. *In vivo* drug evaluation in an animal model can provide more insight into drug action against *P. insidiosum* since it demonstrates not only the direct pathogen-drug interaction (as does *in vitro* assay) but also pharmacokinetic and pharmacodynamic properties of the drug. So far, the rabbit is the primary animal model of pythiosis that has been used for *in vivo* susceptibility analysis. However, the experimental rabbits with pythiosis usually manifest as a subcutaneous lesion, which does not represent the clinical features of pythiosis in humans and animals [31]. Recently, a mouse model of pythiosis has been developed, and it shows similar clinical features of vascular and disseminated pythiosis observed in humans [93]. Thus, the mouse is an alternative animal model for *in vivo* drug susceptibility testing against *P. insidiosum*.

In conclusion, the management of pythiosis is problematic due to the lack of effective treatment. The drug susceptibility testing provides clinically-useful information that can lead to proper drug selection and combination against *P. insidiosum*. Based on the susceptibility results, several antibacterial and antifungal drugs exhibited a profound anti-*P. insidiosum* effect. Some distinct compounds, antiseptic agents, essential oils, and plant extracts, have shown anti-*P. insidiosum* effect. Future studies should emphasize finding a novel and effective anti-*P. insidiosum* drug, standardizing *in vitro* susceptibility method, as well as correlating drug susceptibility data and clinical outcome of pythiosis patients for a better interpretation and application of the susceptibility results.

## Declarations

### Author contribution statement

All authors listed have significantly contributed to the development and the writing of this article.

### Funding statement

This work was supported by Faculty of Graduate Studies, Mahidol University, Thailand (H. Yolanda); Section for Translational Medicine, Faculty of Medicine, Ramathibodi Hospital, Mahidol University, Thailand (H. Yolanda); School of Medicine and Health Sciences, Atma Jaya Catholic University of Indonesia, Indonesia (H. Yolanda); Thailand Research Fund, Thailand (Grant numbers: RSA6280092 [T. Krajaejun]); and Faculty of Medicine, Ramathibodi Hospital, Mahidol University, Thailand (Grant number: CF_61007 [T. Krajaejun]).

### Competing interest statement

The authors declare no conflict of interest.

### Additional information

No additional information is available for this paper.
